# Sociological genealogy of a non-teleological concept of evolution

**DOI:** 10.3389/fsoc.2024.1354362

**Published:** 2024-02-19

**Authors:** Josetxo Beriain, Javier Gil-Gimeno, Celso Sánchez Capdequí

**Affiliations:** I-Communitas, Institute for Advanced Social Research, Public University of Navarra, Pamplona, Spain

**Keywords:** evolutionist evolution, evolutionary evolution, culture, cognition, social fact, non-teleological perspective

## Abstract

The aim of this article is to carry out a sociological-conceptual genealogy of the evolutionist perspective (non-teleological) of approaching social reality. While during the first phases of modernity, a teleological and progressive conception of evolution was imposed, clearly manifested in the proposals of Auguste Comte or Herbert Spencer, in the last decades important bifurcations, processes, and developments have emerged that question the linearity and the finalist character of these positions. We consider that these approaches are closer to the nature of change and social phenomena, so it seems important to us to analyze some of the most outstanding contributions—in the form of sociological genealogy, as we have already mentioned—that have developed this perspective. In order to carry out our task, we have organized four sections: In the first, we make a critique of the sociological evolutionism represented by Comte, Spencer, and Parsons, focusing on the limits of their proposals and the blind spots associated with them. Second, we will analyze the anti-teleological cognitive approaches of Donald and the importance they attach to cultural transmission as a key element for understanding the evolution of both cognition and human societies. In a third moment, we will analyze the coexistence in Weber’s work between the dynamics of ‘disenchantment’ and ‘re-enchantment’ of the world in modern societies, understood as the two sides of the same coin that are in constant dynamic tension and that break with the evolutionary vision that goes from magic through religion to science, or from belief to knowledge. In a fourth moment, we analyze the relevance of approaches focused on what we have called ‘multiple evolutions’ (plural) that collide with each other—the conflicting simultaneity of the non-simultaneous—of their rhythms and directions, inspired by the works of Knöbl, Koselleck, Luhmann, Rosa, Eisenstadt, Abbott, and Zerubavel, which pave the way for the construction of a non-teleological approach to evolution.

## 1 The preparatory narrative of sociological evolutionism^1^ in Auguste Comte, Herbert Spencer, and Talcott Parsons

During an important part of the development of the human and social sciences, the historicist perspective was imposed, which amounted to believing in the existence of ‘historical laws,’ in a kind of ‘law of historical development’, in the existence of an evolutionary pattern in history, even in the orientation of history towards an end. In this scenario, the central task of social science consisted of the discovery, or revelation, of such a law (Popper, 1962).

We will try to explore this idea in the work of the evolutionary sociologists Comte and Spencer, as well as in that of the neo-evolutionary Parsons. For Comte, as for any believer in ‘progress’, the development of humanity has a meaning. In his case, this is articulated as a long road towards the ordered, peaceful, and progressive society of the future, founded on science and presided over by its scientists and wise men (Giner, 2001). In industrial society, wise men come to replace priests and theologians as a social category that provides the intellectual and moral basis of the social order. These social figures would come to represent the predominant way of thinking and the ideas that—in Comte’s opinion—serve as a principle to articulate and develop the social order. In the same way that scientists or wise men are poised to replace priests and industrialists, in the broad sense of the term—that is, businessmen, factory managers, industrial workers and technicians, and bankers—these figures are poised to take the place that warriors played in traditional society. Comte does not foresee anything beyond this state of plenitude, a situation that, according to him, synthesizes all the previous ones and includes all their achievements. This is how he puts it in his *Course in Positive Philosophy*: “It is up to positive philosophy alone to finish what it alone began as the necessary and continuous evolution of an inevitable and spontaneous development whose final direction and general march are exactly determined by fully natural laws” (Comte, 1981, p. 58). In this scenario, the previous phases “constitute true *progress* as an indispensable preparation for the most advanced regime [represented by the positivist-industrial phase]” (Comte, 1981, p. 56, 76). The necessary and continuous progress acquires its fullness in the positivist phase (Comte, 1981, p. 57, 106). Further on, he abounds in the same idea: “The true positive spirit consists, above all, in *seeing in order to foresee*, in studying what is, in order to conclude from it what will be in conformity with the general dogma of the invariability of natural laws” (Comte, 1981, p. 72, 75).

These reflections announce that movement, which serves as a preparatory narrative and which reveals a change in the semantic structure of historical becoming in the sense that the self-contained and self-created ‘perfection’ inherent in God is historically extended in the form of ‘intramundane perfection,’ ‘perfectionement,’ ‘perfectibilité,’ ‘Vervollkommung,’ ‘Vervollkommlichkeit,’ both of the individual and of society. ‘Progress’ is understood here no longer as a concept of religious hope but as the consequence of the action of the individual in society, even in its unintended aspects. The sense of ‘progress’ that derives from this meaning is that of a movement-oriented towards an improvement of the present situation. This is made clear by Nicolas de Condorcet in his *Esquisse d’un tableau historique des progrés de l’esprit humaine* (1980 [1795]), for whom the improvement (amélioration) of the human species must be treated as an endless process since such improvement has no limits other than those of progress itself:

“Finally, will the human species improve, either by new discoveries in the sciences and in the arts, and, as a necessary consequence, in the means of particular well-being and common prosperity, or by progress in the means of conduct and in practical morality, or, finally, by the actual improvement of the intellectual, moral and physical faculties, which may also be the consequence, either of the improvement of the instruments that increase the intensity and direct the use of these faculties, or even of the improvement of natural organization?” (de Condorcet, 1980, p. 226).

A little further on he adds: “Would it be absurd to suppose now that this perfection of the human species must be considered as susceptible to indefinite progress, that a time must come when death will be nothing but the effect, either of extraordinary accidents, or of the slower and slower destruction of the vital forces, and that, finally, the duration of the average interval between birth and this destruction will have no assignable term either?” (1980, 247). As we can observe, expressions such as ‘increase,’ ‘growth,’ ‘development,’ ‘improvement,’ ‘unfolding,’ ‘formation,’ among others, reflect certain uses of progress that orient towards the understanding of progressive innovations as improvement, as hope for something better, or as a process, as becoming. In the opinion of Raymond Aron (2013, p. 76), in the Comtian approach, there are three ideas that agglutinate and allow us to summarize his contribution:

- The society that is beginning to develop in the West is an example to follow; all humanity will have to advance along the path followed by the Western vanguard. That is, the West as a guide to progress and evolution.
- The history of humanity is the history of the spirit as the unfolding of positive thinking, or even of the learning of positivism by all men.
- Human history is the development and flowering of ‘human nature.’

Second, Spencer takes as a starting point the macro-Universal Law of Evolution, according to which “we propose in the first place to show, that this law of organic progress is the law of all progress. Whether it be in the development of the Earth, in the development of Life upon its surface, in the development of Society, of Government, of Manufactures, of Commerce, of Language, Literature, Science, Art, this same evolution of the *simple* into the *complex*, through successive *differentiations*, hods throughout. From the earliest traceable Cosmical changes down to the latest results of civilization, we find that the transformation of the *homogeneous* into the *heterogeneous*, is that in which Progress essentially consists…” (Spencer, 1972, p. 40, Italics from ours). From this perspective, there would not be evolutions in the plural, but a single evolution that permeates all reality in an absolute way: “While we think of Evolution as divided into astronomic, geologic, biologic, psychologic, sociologic, and so on, it may seem to some extent a coincidence that the same law of metamorphosis holds throughout all its divisions. But when we recognize these divisions as mere conventional groupings, made to facilitate the arrangement and acquisition of knowledge when we remember that the different existences with which they severally deal are component parts of one Cosmos; we see at once that there are not several kinds of Evolution having certain traits in common, but one Evolution going on everywhere after the same manner” (Spencer, 1972, p. 72).

Spencer believes in progress, in the organic progress that develops through differentiations: “It will be seen that as in each event of to-day, so from the beginning, the decomposition of every expended force into several forces has been perpetually producing a higher complication; that the increase of heterogeneity so brought about *is still going on, and must continue to go on; and that thus Progress is not an accident, not a thing within human control, but a beneficent necessity*” (Spencer, 1972, p. 52, Italics from ours).

Moreover, this progress has a direction and an end: “Social progress is supposed to consist in the production of a greater quantity and variety of the articles required for satisfying man’s wants; in the increasing security of person and property; in widening freedom of action: whereas, rightly understood, social progress consists in those changes of structure in the social organism, which have entailed these consequences. *The current conception is a teleological one*. The phenomena are contemplated solely as bearing on human happiness. Only those changes are held to constitute progress which directly or indirectly tends to heighten human happiness. And they are thought to constitute progress simply because they tend to heighten human happiness” (Spencer, 1972, pp. 38–39, Italics from ours).

However, events such as the growing militarism of the Victorian era in England (and in other countries such as, for example, Prussia) forced Spencer to modify his initial tendency (shared with other thinkers of the time) to understand evolution as something linear and unique. His patient accumulation and collation of ethnographic and historical data that were arriving in Europe from all over the world also made him see -at the end of his life- that the tendency towards complexity, to the functional specialization and to the civil and industrial way of life, not was as universal as he had originally thought, and that each society or people followed different routes in their way to evolution, some of which would be truncated paths (Giner, 2001, p. 163). We will explore these routes since we cannot affirm that there is a law of historical development leading to a final stage, but rather, as Koselleck (1979) or Dobry (2000) remind us, the *conflicting simultaneity of evolutionary rhythms in dynamic tension*.

The neo-evolutionist thinker Parsons will offer us a third linear and irreversible evolutionist perspective on the universalist character of societies. Parsons’ evolutionist scheme entails greater complexity than those of Spencer and Comte, but it still depends on that normative component that situates *advanced modernity* as the *telos* of a long evolutionary journey that finds its realization in the process of advanced modernization of the United States as the most all-encompassing step of a civilizing mission. In short, Parsons provides a cybernetic model for the cultural direction of change that emphasizes four processes (differentiation, adaptive enhancement, inclusion, and value generalization) but is subordinated to a theory of social order (Joas and Knöbl, 2016, p. 75). For Parsons, a social system tends towards a ‘stable equilibrium’, a lasting preservation of itself as a system and the maintenance of a certain structural pattern, whether static or dynamic. It is analogous (not identical) to an organism and its tendency to maintain equilibrium, or ‘homeostasis’. In this model, the conservation and transmission of norms and values, and thus any theory of action, are linked to a theory of order. Any emergence of value conflict is seen as a deviation from norms and a set of instituted values.

What he considers the inevitability of modernity makes him impute to Weber an evolutionist label, which in reality does not correspond to the Weberian proposal. As Cohen et al. (1975, p. 240) and Morcillo Laiz and Weisz (2016, p. 26) have clearly seen, we can apply to Parsons what Weber criticized Stammler for: that he confuses the normative regulation of behavior by means of rules with the factual regularities of human behavior. Science is also conditioned by society and its value conflicts. This is something that Weber has very present in his model, but that Parsons forgets, neglecting the role of history and its contradictions and contingencies.

## 2 Merlin Donald’s non-evolutionary evolutionary model of cognition and culture

Donald (1991) has established the master lines of an evolutionary process that begins with the mimetic culture two million years ago with the *Homo erectus*, continues with the mythical-symbolic culture that begins approximately 250 thousand years ago, and leads to the theoretical culture that finds its maximum expression in the Axial Era 2,500 years ago. Donald (1991) and Bellah (2011, pp. 265–282) have developed *an evolutionary, non-evolutionist model* with *a clear anti-teleological, anti-finalist armor*, which allows to overcome the inadequacies of a stages model that tends towards an endpoint as it happened in evolutionist authors such as Comte or Spencer and neo-evolutionists such as Talcott Parsons. We assume an evolutionary perspective, but not an evolutionist one. What we mean is that both natural evolution and socio-historical evolution are not directed to an end with a preset script, as, for example, Marx thought, but rather that in both there is fortune, accident, chance, divine Providence, contingency, uncertainty, and the “invisible hand,” which continually force us to rewrite the script and make the existence of an end unfeasible. We usually think that the mimetic phase of our development as human beings is surpassed by the symbolic phase, where we construct images and symbolic representations of reality, and that this phase is surpassed in the conceptual-theoretical phase, where abstract thought makes a tabula rasa of all that has gone before, but this does not happen; *a new stage supposes rather a reconfiguration of old and new possibilities, instead of a surpassing and disappearance of the previous stages*. The interesting thing about Donald’s model, as opposed to all historicism or enlightened teleologism, is that it allows us to understand the evolutionary phases without a finalistic bias where the theoretical culture would have eradicated the mimetic and mythical developments.

Our brains have retained vestiges of our evolutionary ancestors. The nervous system of vertebrates (fish, for example) is very old, and we have retained elements of the vertebrate brain, especially in the organization of the spinal cord and medulla oblongata. A radical change in evolution occurred in the transition from the aquatic to the terrestrial environment. New “modules” emerged in order to cope with the more complex needs of this environment in the form of hypothalamic, basal ganglia, and cortical “modules” present in the mammalian brain. Changes in brain structure among mammals are related to size rather than to the appearance of new structures. There was a large growth in the size of the cerebral cortex between higher mammals and monkeys. But the difference between an ape brain and a human brain is again one of size. Comparing these three brains, we find that the size of the primary cortical areas (those in charge of sensory-motor functions) is similar, in principle, but, in higher species, the secondary and especially the tertiary cortical areas (those in charge of sensory-motor processing) are the ones that experience the greatest increases in size, especially in the human species. In other words, we have conserved a good number of brain structures throughout evolution, even though we seem to have developed others, especially in the cortex (Donald, 1991).

In human beings, the factors that determine the anatomy of our cerebral cortex are genes, environment, and enculturation (Donald, 1991, pp. 355–360). For example, the structure of the basic computational unit of the cortex is genetically established. However, the connectivity between cortical columns that brings with it great computational power, based on experience, depends on the environment, especially in the fundamental stages of development. Moreover, the process of enculturation determines the plastic anatomical changes that allow a whole set of circuits to be integrated into the daily human performative capacity. This can be demonstrated experimentally. Genetic mutations lead to drastic functional deficits, but if there is no genetic problem, limited exposure to the environment (e.g., covering one’s eyes with a blindfold during critical phases of development) can lead to lifelong deficits (blindness). If not exposed to the influence of enculturation, then symbolic skills and language do not develop, leading to dramatic effects on the individual.

The unprecedented development of the cortex exposed to culture allowed for the development of more complex skills, language, and an unparalleled human performative capacity. It is thanks to this, to our ability to acquire symbolic skills, that has led to our superior intelligence, yet research in recent decades rightly highlights a brain–environment co-implication that has given rise to a conclusion in neurobiology: the brain results from a long *epigenesis*, which means that habits, experiences, and education play a determining role in the formation of neural connections. The latter brings with it a different way of considering brain dynamism and the dynamism of cultural artifacts created to process, store, and actively function in the production of collective knowledge.

Once we add symbols, alphabets, and logical-mathematical formulations, biological memory becomes inadequate to store collective knowledge. In other words, the human mind becomes a “hybrid” structure, only partially built from vestiges of previous biological stages with new brain dimensions, widened or very dynamic, according to an adaptive and evolutionary “neuronal architecture” that can even quickly simulate virtual and unlimited operations.

Thus, we now understand how memory devices such as museums, libraries, books, computers, etc., which, for their part, have altered mental organization, i.e., the way we “think” (Donald, 1991), lead to the challenging presence of simulating technologies that displace the centrality of the human brain.

The ‘hardware’ that contributes to the deployment of this new adaptation is no longer biological but technological and is supported by graphic invention, theoretical construction, and the deployment of an external memory. The human brain has co-evolved along with its cognitive cultures over more than two million years but has reached a point where the ability to adapt to specific environments appears as a key element in brain plasticity and therefore in behaviors, cultural forms, and objects. It seems then that it cannot realize its design potential outside of culture and relationships with whatever its environments may be. The mind has become less and less fixed on the neocortex and more on the possibilities of an *acting subject*, in contrast to a mere adaptive attribute to given circumstances, without which even today’s posthumanism would cease to be a critical discourse. As we co-evolve, we act within ‘cognitive collectivities’ in symbiosis with external memory systems such as museums, libraries, temples, monuments, smartphones, computers, and so on. This transformation has led to one of the greatest reconfigurations of cognitive structure without major genetic changes in the history of mammals—indeed, our genes are virtually identical to those of a chimpanzee or a gorilla, but our cognitive architecture is not. It is inextricably linked to these ‘cognitive collectivities’. We have become more complex, multi-dependent, hybrid minds, carrying within us, both as individuals and as societies, all the evolutionary heritage of the past two million years. That is, to the extent that we use our mental abilities to continue developing technology, this technological enculturation has an impact on the way we process information and on the way our brain is shaped. This implies that we are more complex than any previous creature and that we may not have reached our final evolutionary form. *We continue to evolve. We evolve in the way we evolve*. The bottom line is that a series of converging technologies: nanotechnology, biotechnology, artificial intelligence, and Big Data (NBIC) redefine the boundaries of what the humanist position and humanism have hitherto considered human nature.

## 3 The dynamic tension between ‘disenchantment’ and ‘re-enchantment’ of the world as two sides of the same coin in Max Weber

In this section, we will outline the sociological profiles of an alternative interpretation of the concept of ‘disenchantment of the world’ coined by Max Weber, whose wide reception in the social sciences has tended to be interpreted with a finalist bias, ranging from magic to science and from the religious to the secular sphere. Similarly, Weberian ‘disenchantment of the world’ has also been associated with a bias of irreversibility that places the scientific narrative as dominant, making a *tabula rasa of* the previous mythical-symbolic cultures and mimetic culture. The new concept of ‘disenchantment of disenchantment’ that we propose analyzes the co-presence of ritual, symbol, and reason in modern society in such a way that *disenchantment and re-enchantment of the world* become two forces that coexist at the same time. We must keep in mind that the ancient sacred forms of universal religions have not died (contra Nietzsche) and yet, at the same time, new self-sacralizations of secular spheres such as, for example, the king, the nation, and the human person have acquired the status of transcendence in modernity. As Weber himself pointed out, the old forms continue to compete with each other and also with the new forms of sacralization, that is, with the new disenchanted gods of the secular orders, in a never-ending struggle.

The *disenchantment of the world*, a multifaceted concept analyzed mainly by Weber as an invisible social force, has created a canonical narrative and a strong research program throughout the social sciences. Disenchantment of the world underlies *background* concepts such as differentiation, rationalization, and modernization without being assimilated point-by-point with any of these.

Initially, it is important to delimit *what disenchantment of the world is not* before being able to affirm what it can be. It is not the end of belief in magic. It is not the end of belief in certain types of animate, mysterious, or supernatural beings, as Émile Durkheim had already warned at the beginning of *The Elementary Forms of Religious Life* (1982) in 1912. It is not a new pessimistic fashion, nor is it the fragmentation of socio-symbolic cohesion. It does not represent the emergence of instrumental reason because magic itself is instrumental and demands a certain degree of rationalization. It does not yet represent secularization, insofar as *disenchantment of the world* is prior to and within religion itself. It is not the evolution from magic to religion and from religion to science, as authors like Frazer would understand it, because Weber repeatedly reminds his readers that magic and religion often coincide.

What, then, does *disenchantment of the world* mean—*demagicization*? Obviously not, because magic and myth are still present everywhere in plural form. *Disenchantment of the world* as *desacralization*? Neither, because we face day after day the emergence of new sacred realities as each society produces its own sacred forms. From Durkheim (1982) to Alexander (2003), we know that each society creates its own sacred realities, no matter whether these are pre-axial (nature, mana), axial (the gods), or post-axial (the nation, people, the human person, humanity). *Disenchantment of the world* as *Detraditionalization*? Yes, because the new (polytheistic) form of the specifically modern constellation of values is different from the old (monotheistic) one. The more disenchantment the secularized cultural value spheres of the world become more differentiated and autonomous, thus creating a polytheism that, in the public sphere, manifests itself as a Culture War (*Kulturkampf*) and, therefore, finds it more difficult to develop a rational integrated intramundane way of life that gives ‘meaning’ to life as a whole. In other words, in ‘disenchanted’ societies, the emergence or development of what we know as a unitary ‘ethical personality’ becomes more complicated. *Disenchantment of the world* as *Secularization*? Not as the only encompassing and dominant trend of value change. In fact, Weber hardly ever used the word secularization in his writings.

On the one hand, the *disenchantment of the world* appears in the “Sociology of Religion” included in *Economy and Society* (2002) as a *historical-religious* process that supposes a rationalization of religious worldviews in which, increasingly, “the world as a fallen creature has religious meaning only as an object of realization of duty conducted by a rational action, according to the will of God that projects itself sovereignly over the world” (Weber, 2002, p. 438); but on the other hand, according to the “Excursus” of 1920, *disenchantment of the world* would be a *historical-scientific* process according to which, once empirical-rational knowledge realizes *disenchantment of the world*—transforming it into a causal mechanism—then a tension appears against the ethical postulate according to which the world is a universe governed by God that carries with it a soteriological and ethical meaning. The empirical foundation of the world, as well as the mathematically oriented one, clashes with any conception of the world that is oriented in some integrative sense (Weber, 1998, p. 553). In “Science as a Vocation,” Weber expresses himself in similar terms:

“Increasing intellectualization and rationalization *do not* mean, then, an increasing general knowledge of the general conditions of our life. Their meaning is quite different; they mean that one knows or believes that at any time one *wants*, one can come to know that, therefore, there are no hidden and unpredictable powers around our life, but that, on the contrary, everything can be *mastered by calculation and foresight*. This simply means that the magical has been excluded from the world” (1996, 199–200) (see footnote 1).

According to this reasoning, the sociological tradition has mixed both characteristics of the concept in an encompassing narrative with a canonical tenor, in which a bias of irreversibility has been constructed, as well as a teleological tendency that goes from magic to science, from the sacred to the profane, from the religious to the secular. Today we have at our disposal relevant sociological analyses^2^ thanks to which we can face with guarantees the realization of a sociological genealogy of disenchantment of the world, understood as *a* sort of *disenchantment of disenchantment itself that would be able to explain the* var*ieties of re-enchantments and sacralizations that arise, sometimes as an undesired consequence in relation to the disenchantment of the world itself in the form of a transit from Judeo-Christian monotheism to the modern polytheism of the ‘new gods’; and at other times against the process of disenchantment of the world itself, as happens when the emerging narrative of the sacralization of the human person—coming from human rights—clashes with the dominant narrative of the nation*.

Therefore, the process of social evolution is neither irreversible nor teleological; it is open and subject to multiple contingencies. Weber’s ‘modern struggle among the gods’ or the so-called ‘modern polytheism’ is not the end of social differentiation in the same way that *disenchantment of the world* is not the end of the evolution of religions. Disenchantment and re-enchantment of the world are two sides of the same coin. The evolutionary ‘overcoming’ of magic is highly improbable. Each new stage of evolution forms a new constellation of relations between the old and the new, the sacred and the profane, magic and science, as we have seen in the previous epigraph, but it does not imply something like *a finalistic passage from one type to the other*. There is no evolutionary logic that goes from ‘enchantment’ to ‘disenchantment’ but *a field of (inter-)action in which there are dynamic tensions between the two processes over time*.

## 4 Multiple evolutions, their rhythms, and directions

Having analyzed, on the one hand, the teleological evolutionary perspective and, on the other, the non-teleological evolutionary perspective, and having placed the Weberian ‘disenchantment of the world’ in a non-teleological evolutionary interpretative context, we then develop a series of avenues of sociological analysis (four to be precise) that arise from the ‘disenchantment of disenchantment’, and which place us in a scenario of ‘multiple evolutions’, a concept that we will try to define briefly in the conclusion.

### 4.1 The dynamic tension between teleological and anti-teleological narratives within modernity

Wolfgang Knöbl (2022: 141 ff.) openly raises—with theoretical and empirical arguments—the plausibility of positing an anti-teleological model of evolution that is inspired by the work of Toulmin and Goodfield (1965). Knöbl asks a couple of relevant questions regarding a non-teleological conception of evolution: The first of these concerns the preconditions that had to be met in order to think of *natural* history in the way Darwin had proposed it in the second half of the nineteenth century in a strictly anti-teleological way, and the second question interrogates *human* history in an equally anti-teleological way, something that was anything but self-evident in the nineteenth century and remained anything but self-evident also in the second half of the twentieth century. Toulmin and Goodfield comment that, in the beginning of Greek historical descriptions and in Greek philosophy itself, there were attempts to think of history as a development; however, the great influence of the proposals of Plato and Aristotle caused these ideas to be buried, considering that the changing, the non-constant, the accident—as opposed to Substance—represent something that can be transformed in the future and, therefore, cannot shape the essence of reality understood as something immutable. In this historicist-idealist perspective, the series of historical events would be nothing but indicators that serve to explain the texture of that *reality that remains unalterable*. This idea is somewhat untenable for us since we have been socialized within a new social ontology where accident, contingency, and uncertainty act as constitutive principles of social reality, thus opening an *anti-teleological horizon* within the human and social sciences, as is made clear in this extensive fragment we quote from Toulmin and Goodfield below:

“After the establishment of modern historical criticism and Darwinian theory, it would be naive to continue to assume that history represents a *single* process or with a demonstrable *direction*. To regard the ancient Hebrews, classical Greece and Rome, and Christian Europe as the ‘main route’ of history is too much like, in our view, Lamarck’s habit of selecting certain groups of each paleontological epoch as ‘the spearhead of evolution’ while despising lesser races. The course of history has been much more complex than that, and the continuous interaction between different cultures precludes any possibility of identifying any ‘march of time.’ The same is true of the idea that history has a demonstrable direction: the deeper lesson of Darwin’s work is that new creations of great functional significance often arise as by-products of processes whose manifest goals go in very different directions, and that the merit of these novelties depends, not on their conformity to a long-term historical trend, but on their immediate appropriateness to the particular situation at hand. This is equally true for both agencies and institutions. If there is a key to understanding all history, it lies in recognizing not its unidirectional character, but its multiple opportunism” (Toulmin and Goodfield, 1965, p. 235).

Just as the first modern evolutionist narrative represented by Comte and Spencer created a linear and finalist conception of social evolution whose stage of plenitude was the industrial society of 1848, one hundred years later—from 1945 onwards—a continuist narrative emerges that aims to describe a globality of the processes of ‘modernization’ according to which the processes of transformation of traditional societies into modern societies take place in a relatively uniform and linear manner. Levy, Walt Rostow, Seymour Martin Lipset, Gabriel Almond, Sidney Verba, and Edward Shils are the builders of this narrative. In Knöbl’s opinion, these would be some of the most relevant milestones that characterize this new narrative (Knöbl, 2022, p. 147 ff.):

First, history appears as a *history of progress*, as a progressive history. Of course, there are differences in the development process from one country to another, but what remains unchanged is the *pathway* that guides the development of all of them. If before the model was that of the British commercial and industrial society of 1848, now the model will be the post-war United States (Ekbladh, 2011). It is no longer enough to speak of modern society as the *telos of* social development; rather, this development is doubly adjectivized as *advanced modern* society, within a logic of irreversible stages of development. Nevertheless, the very differences in the processes of development already show a *multiplicity* (Ramstedt, 1975, pp. 47–63) in which there are models that emphasize the experience of ‘occasional’ time; in other cases, the temporal experience is ‘cyclical’; in other cases, there is a ‘linear temporal consciousness with a fixed future’; and, in other cases, the temporal experience indicates a ‘linear consciousness of time with an open future’. All this brings out the dynamic tension that we have mentioned between the teleological model and the non-teleological models.

Second, the concept of ‘modernization’, in principle, is equated with the notion of social change, within which there are sub-processes such as industrialization, rationalization, individualization, or democratization that have their own rhythm, speed, and direction, thus making the model more complex. It becomes increasingly difficult to speak of the linearity of the model and of synchronized parallel developments, as each sub-process, each sub-system, has its own time, as Luhmann (1997, 1998) has shown.

Third, in the model of ‘modernization,’ which is considered as the ‘traditional,’ the ‘non-modern’ appears as a residual category as opposed to the category of the ‘modern.’ In this binomial, in the *foreground*, the two elements of the aforementioned pairs—traditional versus modern—are logically comparable, although, however, in the *background*, they are cognitively and morally unequal, by implicitly carrying a process of *cognitive hegemonization* (Zerubavel, 2018) of one pole (modern) at the expense of the other (traditional); the ‘normalization’ of one pole produces the ‘a-normalization’ of the opposite pole, and vice versa. History confirms this if we approach the analogy drawn in the nineteenth century between the successful England of the First Industrial Revolution and the Egypt of Ramses II, something that appears both in the writings of the representatives of the Scottish Enlightenment, in Hegel (2010) himself and, also, in Marx and Engels (2019)—and Engels—when he states that the ‘communist society’ represents the authentic realization of modern industrial society. Perhaps the historian Lucien Febvre already put us on this track when, in 1950, he wrote about another French historian, Jules Michelet: “The Renaissance would not have been, beyond a senile flexion, but a resurrection of the original Middle Ages, of the Middle Ages in their first purity of the true Middle Ages in what it had of better… But Michelet’s Renaissance was not a restitution of medieval purity. It was the negation of the Middle Ages. It was a rupture of tradition. It did not add a link to the chain. It came out of nothing. *Tabula rasa*. Or, if you prefer, *Miracle*. Michelet has put it in his own way, magnificently: ‘The heroic cast of an immense will’” (Febvre, 1992).

Fourth, there has been a shift from the *adjectivization of the* main constituent elements of society as *modern*—thus, modern bureaucracy, modern capitalism, modern religion, modern personality, modern communication, and so on—to their *substantivization*, indicating an important change of semantic emphasis. It seemed indisputable that all the macro-historical processes considered central would run towards this modernity—from democratization to secularization, passing through individualization. On the one hand, ‘modernity’ would thus constitute a kind of anchor point, a position that could hardly be questioned and therefore could not be historicized, from which world events to date could be analyzed and which obviously also possessed the charm of coupling itself—to a certain extent—to a global concept of change, that of “modernization” (Knöbl, 2017), and to its teleological drift. But, on the other hand, the new emerging social reality generates the very conditions of possibility for the *critique* of linear and finalist assumptions (Boltanski, 2014), as Baudelaire makes clear when he affirms the *ambivalence* ascribed to modernity: “Modernity is *the transitory*, the *fugitive*, the *contingent*, half of art, where the other half is the eternal and immutable” (2000: 92).

### 4.2 The conflicting simultaneity of the non-simultaneous

Undoubtedly, one of the most relevant anti-teleological critiques is that of Koselleck (1979, 2000). According to him, the experience of subjects after the political and social revolutions implies a *denaturalization of the experience of time*, since we live in a new time, a time that is no longer simply the medium *in* which all histories take place but that gains historical quality. Consequently, history (or histories, plural) no longer takes place *in* time but through *it, for the sake of* time^2^. Time, as such, becomes a historical and dynamic force. In its semantic course, the nature of Fortune as ‘daughter of the divination of the future’ or as ‘mother of chance’ that served to justify the repetition of a transpersonal set of events beyond the control of men and women, as soon as it is interpreted empirically or pragmatically thanks to the rationalization carried out by Aristotle, becomes pure chance, opportunity, accident (Koselleck, 1979: 160 et seq.), the object of rational planning. Chance becomes a ‘motivational remainder’ for action. The facts, although they may be rationally grounded, remain contingent, but the difference is that they arise in a space of human freedom. There is a glimpse of the possibility that human will can control contingency. For Koselleck, the experience of time in modernity is expressed as a *growing difference* between the ‘space of experience’ (the past) and the ‘horizon of expectations’ (the future). As he himself comments: “In modern times, the difference between experience and expectations has increasingly expanded, more precisely, modernity has been understood as a ‘new age’ since expectations have been increasingly distanced from all previous experience” (Koselleck, 1979, p. 359). Thus, in front of the concepts of History and Progress (in singular) understood as central categories of modern self-understanding, there also appear histories and progress (in plural) in science and technology, in morality and art, in law, in politics, in economy, and so on, as a consequence of the unstoppable process of functional differentiation of social systems that configure not only a new time (*neue Zeit*), but the *newest time* (*neueste Zeit*), understood as a condition of possibility for any self-understanding of modern societies. The rapid acceptance of such a form of ‘newer time’ is “an indicator of the *social acceleration* in the rate of change of historical experience and of the increase of a consciousness of time acting upon itself” (Koselleck, 1979, p. 279). Undoubtedly, the *acceleration of time* (Koselleck, 2000, p. 150 ff.), understood as an increase in the speed of movement of messages, people, and goods, is going to be one of the great conditioning factors of the experience of the subject in modern life. Although it may be difficult for us to accept it, the inexorable fact—beyond all romantic nostalgia—is that “we live in a world that is no longer based so much on geographical extension as on temporal distance—in the space of time—constantly reduced by the capacities (discovered and deployed) of transport, transmission and tele-action” (Virilio, 2001, p. 84).

The experience of acceleration has two consequences: on the one hand, a “*contraction of the consciousness of the present*” (Lubbe, 2003, p. 399 ff.), as an effect of social acceleration, which in many cases manifests itself as a “tyranny of the present” (Eriksen, 2001, p. 41 ff.), of the moment, expurgated of any burden of tradition and of any utopian conception; and, on the other hand, the *experience of the simultaneity of the non-simultaneous*. Undoubtedly, the latter is Koselleck’s strongest and most empirically tested critique of the teleological conception of modernity. The ‘non-simultaneous’ means that qualitatively different stages of development appear ‘simultaneously’ within the same quantitatively measurable time (clock time, abstract, universalized, with its time zones). This contrast has several roots. The first of them refers to the confrontation that takes place at the end of the fifteenth century between the European culture that interprets itself as the most advanced world culture and the Mesoamerican cultures of the ‘new world’, interpreted by the former as primitive and less developed (Koselleck, 1979, p. 290). In this new world model, social processes have their *own* temporal structure, as Herder makes clear: “At present, every changing thing carries with it the measure of its own time (…) There are innumerable times in the universe” (Herder, 1995, p. 68). The geographical opening of the globe (the discovery of ‘new’ geographical zones) brought to light a variety of coexisting ‘cultural levels’ that, through processes of synchronic comparison, were diachronically ordered (Lévi-Strauss, 1975; Bestard and Contreras, 1987, pp. 15–38, 49–70, 84–92; Chakravorty, 2000; Bhattacharya, 2011). Thus, the contemporaneity of the non-contemporary (‘backward,’ ‘underdeveloped,’ ‘barbarians,’ ‘savages,’ ‘primitives,’ ‘pagans’) *participates*—albeit in an unequal way—in the new myth of ‘progress’. Within this new spatiotemporal context that defines the imaginary meaning of progress, *different rhythms* (more or less accelerated) of social-historical change are configured, all of them sustained around constellations of meaning of the metropolis colony, capitalism development, and socialism dependence revolution type, which denote the selective links existing between Western nation-states and their global environment. In an influential essay entitled *Time and the Other* (1983), the Dutch anthropologist Johannes Fabian considers that modernity was born when the timeline established by Grafton’s chronologists was spatialized across a vast geo-chronocultural slate encompassing the entire planet. This timeline functioned as a concentric secular cosmology that grouped all the peoples of the planet into a new *world map*, with the great cities of Europe as the new Jerusalem, being treated as the origin and summit of civilization and as the only part of the planet that was actually *modern*. There was a powerful new form capable of making sense of the flow of discontinuous, fragmentary, and destabilizing evidence about human origins and habits that was pouring into the enlightened societies of these cities. This cosmology of modernity is founded on the original sin of a hegemonic ambition, the ‘denial of contemporaneity’ of all those allegedly involved. According to Fabian: “Anthropology contributed above all to the intellectual justification of the colonial enterprise. It gave politics and economics—both concerned with human Time—a firm belief in ‘natural’, i.e., evolutionary, Time. It promoted a scheme according to which not only the cultures of the past, but all living societies were irrevocably situated on *a* temporal slope, a stream of Time, some upstream, some downstream” (1983, p. 17). It is not the dispersion of human cultures in space that leads anthropology to ‘temporalize’ (something that is maintained in the image of the ‘philosophical traveler’ whose wandering through space leads to the discovery of ‘ages’), but rather it is naturalized-spatialized Time that gives meaning (indeed, a variety of specific meanings) to the distribution of humanity in space. The history of anthropology “reveals that such use of Time is almost invariably made for the purpose of distancing the observed from the observer’s Time” (Fabian, 1983, p. 25).

A second root refers to the problem of the non-simultaneous within the internally differentiated modern society itself since it must confront the inequality of progress in the different parts that make up its social structure—law, science, art, politics, economy, family, and so on—as well as the existing inequality among men. Koselleck, following Friedrich Schlegel’s arguments, speaks about the simultaneity of the non-simultaneous (Koselleck, 1975, p. 380), understanding as such the confluence of differentiated speeds in the course of history, represented by the different rhythms of both intra-societal and inter-societal change. Friedrich Nietzsche in philosophy, Baudelaire (2000) in literature, Bell (1976), Eisenstadt (1986), Durkheim (1987), Luhmann (1997), and Weber (2002) in sociology have highlighted—with different nuances, of course—the progressive differentiation of social spheres, thereby illuminating the possibility for us to introduce the term ‘society without a center’ or ‘de-centered society’, where there is no longer an instance, much less a supra-social meta-instance, that integrates society as a whole, religiously, politically, or economically. Faith, political power, and money undoubtedly act according to their own intrinsic logics of functioning. That is to say, there is a *constellation of simultaneity* in which different social units each deploy their own speed in different social spheres, lacking an encompassing temporality and futurity, since what we may call progress in one unit manifests itself as retrogression in another. Perhaps the best example of this is the current situation in the European Union. This results in a fiction of unity, making the concept of global planning obsolete. The concept of ‘progress’ that emerges from this situation comes from different sectors and from different units of concrete action between which there is a relationship of *temporal tension*. Therefore, such a concept is partisan in that it is associated with a sphere of action. Instead of singularized progress, we must necessarily refer to the *different pluralized “progresses”* (Valencia, 2007), in many cases asynchronous, that converge simultaneously, or to the notion of a *plurality of futures* (Chakravorty, 2000). Faced with the postulation of a progressive future, singularized and thought of as an object of conquest and the goal of a long process—which has been the aim of a good part of the sociological discourse of modernity and its teleological narrative—we observe the projection of a plurality of futures that concur in the public sphere, forming a war of times that clash among themselves seeking the realization of their own internal legitimacy in future scenarios (Fraser, 1999), as Weber already made clear in his *Zwischenbetrachtung* written in 1920: “Rationalization (…) led to the fact that the specific internal legalities of each particular cultural sphere of value became conscious in their consequences and thereby entered into mutual tensions” (Weber, 1998, p. 441).

### 4.3 Increasing the probability of evolutionary improbability

Luhmann also offers us a non-teleological concept of evolution. According to him: “Traditional concepts of rationality had lived on external advantages of meaning. With the secularization of the religious ordering of the world and with the loss of the representation of univocal starting points, these advantages lose their grounding. Therefore, judgments about rationality have to detach themselves from the external advantages of meaning and readapt themselves to a unity of self-reference and hetero-reference that can always be produced only within each system” (Luhmann, 1998, p. 148). Modern societies are ‘multiple units’. “Society unfolds into different functional spheres” (Luhmann, 1997, p. 743 ff.), into different orders of life such as economy, politics, science, religion, law, sport, and so on. Each of these partial systems configures a specific and proper way of solving problems. There is no universal ‘reason’, but rather sub-specific criteria of rationality: justice, truth, beauty, property, etc. The questions to be answered by this new scenario, therefore, are 2-fold: on the one hand, which system is involved, and, on the other hand, what kind of internal directive distinction does this system use as a device for reducing social complexity? Each system seeks to realize its own directive distinction—having, governance, truth, justice, beauty, and so on—as opposed to its opposite—poverty, ungovernability, falsity of statements, injustice, monstrosity, and so on—but there is no socially guaranteed tendency that assures this because there is no longer a meta-reducer of contingency that assures the process—God—and because contingency is infinitely greater now than a thousand years ago due to the fact that the more we know, the more we know that we know less, due to the multiplication of uncertainty in every sphere of human existence (Luhmann, 1992, p. 37). The more rationally one calculates and the more complex the process of calculation becomes, the greater the number of facets in which the uncertainty of the future reigns. In the social sciences, there is no accumulation of knowledge in the same way; the availability of more knowledge does not lead to more certainty but to more uncertainty (Giddens, 1990, p. 36 ff.). This new context of uncertainty does not refer to ‘the known unknown’, which could be known later through progress, but to ‘the unknown unknown’, to a new kind of evolutionary lack of transparency, to the blind spot from which one observes and does not see that one does not see. The acceleration of historical sequences of events prevents expectations from referring to previous experiences. In this way, “the improbable becomes probable” (Luhmann, 1992, p. 287), since everything, or almost everything, is transformed into an unforeseeable future. In this sense, terms such as ‘greater improbability’ connote a temporal description of states of nature or society. The concept of evolutionary improbabilities refers to the dimension of time. It indicates that it takes time to build systems that presuppose themselves during subsequent evolutions. The arrow of time, therefore, points from more probable states (easy to generate) to more improbable states that feed on previous developments. This description of temporal direction includes a modified notion of progress in the sense that we may (or may not) want to live in or maintain and develop the improbable states in which we find ourselves. This description also includes the ideas of differentiation and complexity in the sense that the modern type of differentiation, namely functional, is a highly improbable state with more negative aspects than segmentation or stratification. The new framework of temporal description encompasses the old ones. Moreover, it also reevaluates them and provides conceptual space to include real feelings of insecurity and risk, distrust in optimizing strategies and good intentions, and inevitable alienation (Luhmann, 1992).

For Harmut Rosa (2005), a society based on acceleration would be one in which the technological dimension (of acceleration itself) and the growth of the *scarcity of time* (i.e., the acceleration of the pace of life) develop in terms of inequality, i.e., if the growth rates of activities to be carried out grow faster than the rates of technological acceleration (Rosa, 2005, pp. 243–255), then time becomes scarce. The more dynamic the environment in which we live and the more complex and contingent are the chains of events and the horizons of possibility configured, the more difficult it is to make compatible the activities we perform and the decisions we make within schedules overloaded with demands of all kinds (Beriain, 2008, p. 111). That is to say, *under conditions of high complexity, time becomes scarce, it is ‘compressed’*. We can express this with a certain sociological conjecture: *the greater the increase in systemic differentiation*, that is, the greater the increase in the need to reduce social complexity expressed in greater ‘social density’, in greater connectivity, the greater the *distance between the past and the future, thus increasing the threshold of generated contingency as opposed to controlled contingency, which is but another way of explaining the concept of ‘probability of evolutionary improbability’*.

### 4.4 Modernity: one, none, or many?

In contrast to the canonical notion of Western modernity that has predominated in sociological analysis, Eisenstadt (2000, pp. 1–31) introduces the notion of *multiple modernities,* which denotes a certain perspective on the contemporary world—on the history and characteristics of the modern era—that stands in contrast to the more usual perspectives represented by the classical theories of modernization and the convergence of industrial societies, predominant in the 1950s. All of them assume, explicitly or implicitly, the cultural program of modernity as it developed in modern Europe, starting in the seventeenth century, and, finally, the basic institutional constellations that emerged because of such cultural ferment, which were imposed on all modern or modernizing societies. Against the conception that considers Western modernity as an all-encompassing concept, which has been the original from which copies have been drawn throughout the world, there is the concept of *multiple modernities* that develop the cultural and political program of modernity in many civilizations on their own terms. Current developments, especially since World War II, in modernizing societies have refuted the homogenizing and hegemonic assumptions of this program of Western modernity. The idea of *multiple modernities* presupposes a new way of understanding the contemporary world—for explaining the history of modernity—seeing it as a history of continuous constitutions and reconstitutions of a *multiplicity* of cultural programs. These ongoing reconstructions of multiple institutional and ideological models are conveyed by specific social actors in close connection with social, political, and intellectual activists and by social movements that seek the realization of different programs of modernity while maintaining very different perspectives on what makes modern societies what they are and what constitutes them.

### 4.5 The process approach

Abbott’s (2001, 2016) processual approach starts with the idea that everything in the social world is continuously in the process of making, remaking, and unmaking itself (and other things), instant by instant. The social world does not consist of atomic units whose interactions obey various rules, as in the thinking of economists. Nor does it consist of large social entities that shape and determine the small lives of individuals, as in the sociology of Durkheim and his followers. Nor does it consist of conflict between given units, as in the work of Marx and his numerous imitators. Nor does it consist of symbolic structures that determine and shape our perception of the social world, as in the tradition following Dolgin et al. (1977) and Geertz (1987). They are all distinguished traditions, and each has its successes in the analysis of human affairs, but they forget that this is a world of *events*. Individuals and social entities are not the elements of social life, but patterns and regularities defined in *sequences of successive events*. They are moments in a temporal course, moments that will shape the next iteration of events, even when they go back in time. In this processual dynamic, there is no beginning or end, only processes. In short, the processual approach is fundamental and essentially historical.

### 4.6 Collective memory or mnemonic communities in plural?

We are much less concerned with what Jesus, Columbus, or Nebuchadnezzar did than with their role as ‘memory figures’ (Halbwachs, 2015). Put differently, we are primarily interested not in what happened in history, but in *how we remember it* (Zerubavel, 2012). Our memories of the past are by no means objective, as we do not all remember them in the same way. However, the fact that these mnemonic battles usually involve entire groups and are fought in unambiguously public forums, such as museums and school boards, seems to suggest that they are not entirely personal.

Mnemonic experience can be articulated in terms of historical *continuity*, but these attempts are often countered by diametrically opposed efforts to create the experience of historical *discontinuity*. And whereas the kind of mnemonic ‘editing’ presupposed by the former is aimed at deliberately glossing over actual temporal gaps between non-contiguous points in history, the latter is specifically designed to help transform actual historical continuums into a series of seemingly independent blocks of time. Instead of a mnemonic ‘pasting’, historical discontinuity implies a mnemonic ‘cutting’, since, instead of attempting to project an appearance of continuity, historical discontinuity implies a mnemonic ‘cutting,’ instead of attempting to project an appearance of emptiness, the goal is to promote a vision of real historical gaps. Chopping up the past into supposedly discrete ‘periods’ is basically a mental act and is usually done with an unmistakable social character. Historical ‘periods’ are basically products of our minds, so *it is very important not to turn our unambiguously conventional periodization systems into essence*.

There are many alternative ways of chunking the past, none of which is more natural and therefore more valid than others. *Any system of periodization is thus inevitably social, since our ability to imagine the historical watersheds separating one conventional ‘period’ from another is basically a product of our socialization into specific traditions of carving up the past*. In other words, we need to be mnemonically socialized to consider certain historical events as significant ‘turning points’. In fact, apart from the Big Bang, it is never obvious at what point a particular stretch of history ‘begins’, and there is always more than a single point that could constitute the formal beginning of a particular historical account.

## 5 Conclusion

Throughout the previous pages, we have carried out a sociological-conceptual genealogy of the evolution of societies that is not articulated on the basis of the assumptions of classical evolutionism, materialized in authors such as Comte or Spencer, but is deployed on the basis of a non-teleological conception of history and of the drift of social action in it, which starts from the fact that, in social evolution, “nothing is ever lost” (Bellah, 2005, p. 72). In this scenario, evolutionary stages neither preserve a teleological linearity nor impose themselves on the past by acting on them as *tabula rasa*. From the perspective we have detailed in this article, each new evolutionary breakthrough implies a reconfiguration of the possibilities of action and choice of the subjects and collectives, in which the features and characteristics of the past do not disappear but are inherited by the subsequent evolutionary forms. All this generates a scenario of complexity that increases as new forms of interaction develop. Hence the notion of multiple evolutions.

This conceptual scheme allows us to approach social reality with more guarantees and helps us to better understand the plurality and multiplicity of forms and social projects that emerge because of evolution. In fact, the unfolding of tensions and features throughout Section 4 is a clear example of how the current academic scenario has detected failures and clear hiatuses between social reality and the explanatory schemes of classical evolutionism. Questions such as those addressed, which call into question the very teleological orientation of evolution through the analysis of the processual logics associated with social and cultural action; which reveal how the non-simultaneous can be experienced in scenarios of simultaneity; how the probability of the improbable can be increased; how there has been an acquisition of awareness of the plurality associated with human behavior and the social action from which those behaviors develop, lead us to the conclusion that any scheme that seeks to understand the evolution of human societies must respect three basic principles inherently ascribed to social action: (1) the principle of continuity with regard to the logics of social construction of reality and culture; (2) the principle of complexity and plurality of forms of social being; and (3) the principle of conflict, understood from the perspective that, as Weber reminds us, there are always dynamic tensions between different and/or alternative ways of understanding reality, between specific socio-cultural programs. The above principles should make us aware that without taking into account the logics of functioning and action in social reality, we cannot understand or articulate a concept of social evolution that allows us to approach with certainty the reality of social evolution. Social evolution is so insofar as it attends to this social condition of evolution, which may be different from other types of evolutionary experiences that occur outside the context of social interaction. It is for this reason that we have argued throughout the previous pages that a scheme that rewards continuity, persistence, and dynamic tension between evolutionary formulas helps us to explain the reality of social evolution much better than other conceptual artifacts that were previously constructed.

## Data availability statement

The original contributions presented in the study are included in the article/supplementary material, further inquiries can be directed to the corresponding author.

## Author contributions

JB: Conceptualization, Formal analysis, Investigation, Methodology, Project administration, Resources, Supervision, Validation, Visualization, Writing – original draft. JG-G: Conceptualization, Formal analysis, Investigation, Methodology, Project administration, Resources, Supervision, Validation, Visualization, Writing – original draft. CC: Conceptualization, Formal analysis, Investigation, Methodology, Project administration, Resources, Supervision, Validation, Visualization, Writing – original draft.

## Footnotes

^1^Evolution represents the set of transformations, of metamorphoses, that affect the different strata of biological, geological, human and sociocultural life, while evolutionism reveals a modern perspective of understanding evolution as a movement that goes through a series of preparatory stages until reaching an end in which evolution supposedly reaches its maturity, its fullness, and which is the object of criticism in this work. The concept of progress would represent that modern evolutionary narrative that finds explanatory and normative plausibility in de Condorcet (1980) and that is critically analyzed, among others, by Koselleck (1975, 1979), Beck (1992), and Latour (2017).

^2^For more information on these issues, see also Debray (2000): *Introduction à la médiologie*, Paris: PUF., and Debray (2018): *Comment nous sommes devenus américaines*, Paris: Folio.
